# *C*-Axis Textured, 2–3 μm Thick Al_0.75_Sc_0.25_N Films Grown on Chemically Formed TiN/Ti Seeding Layers for MEMS Applications

**DOI:** 10.3390/s22187041

**Published:** 2022-09-17

**Authors:** Asaf Cohen, Hagai Cohen, Sidney R. Cohen, Sergey Khodorov, Yishay Feldman, Anna Kossoy, Ifat Kaplan-Ashiri, Anatoly Frenkel, Ellen Wachtel, Igor Lubomirsky, David Ehre

**Affiliations:** 1 Department of Molecular Chemistry and Materials Science, Weizmann Institute of Science, Rehovot 7610001, Israel; 2 Department of Chemical Research Support, Weizmann Institute of Science, Rehovot 7610001, Israel; 3 Department of Materials Science and Chemical Engineering, Stony Brook University, Stony Brook, NY 11794, USA

**Keywords:** aluminum scandium nitride, sputtering, seeding layer, texture, piezoelectric

## Abstract

A protocol for successfully depositing [001] textured, 2–3 µm thick films of Al_0.75_Sc_0.25_N, is proposed. The procedure relies on the fact that sputtered Ti is [001]-textured α-phase (hcp). Diffusion of nitrogen ions into the α-Ti film during reactive sputtering of Al_0.75_,Sc_0.25_N likely forms a [111]-oriented TiN intermediate layer. The lattice mismatch of this very thin film with Al_0.75_Sc_0.25_N is ~3.7%, providing excellent conditions for epitaxial growth. In contrast to earlier reports, the Al_0.75_Sc_0.25_N films prepared in the current study are Al-terminated. Low growth stress (<100 MPa) allows films up to 3 µm thick to be deposited without loss of orientation or decrease in piezoelectric coefficient. An advantage of the proposed technique is that it is compatible with a variety of substrates commonly used for actuators or MEMS, as demonstrated here for both Si wafers and D263 borosilicate glass. Additionally, thicker films can potentially lead to increased piezoelectric stress/strain by supporting application of higher voltage, but without increase in the magnitude of the electric field.

## 1. Introduction

In the search for lead-free, Si-microfabrication-compatible piezoelectric materials, thin films of scandium-doped aluminum nitride (Al,Sc)N are of great interest for use in actuators [1], energy harvesting [2], and micro-electromechanical-systems (MEMS). While the piezoelectric response of AlN increases upon doping with Sc [3,4], difficulties are encountered during film preparation because, as bulk solids with completely different structures and large differences in cation radii, ScN (rock salt, cubic) and AlN (wurtzite, hexagonal) are immiscible [5]. Consequently, (Al,Sc)N is inherently thermodynamically unstable and prone to phase segregation [3]. Film preparation is further complicated by the technological requirement for polar [001] or [00 $\bar{1}$] out-of-plane texture, which is achieved using a seeding layer.

To promote growth of textured (Al,Sc)N films (i.e., Al_1−x_Sc_x_N, x < 0.4), the seeding layer should satisfy at least two requirements: (a) close epitaxial match to the growing film [6,7] and (b) low surface roughness to prevent secondary nucleation [6]. The most common preparation protocol uses reactive DC sputtering onto textured seeding layers of (111) fcc or (110) bcc metals [6,7,8], e.g., Au, Pt, or Mo. These metals are chemically inert towards both $N_{2}$ and (Al,Sc)N, and display lattice mismatch to the (001) planes of (Al,Sc)N of 7.5%, 10.8%, and 12.4%, respectively [6]. It was observed that even when growing films of (Al,Sc)N initially display strong [001] texture, orientation is often lost once the film thickness exceeds a few hundred nm [3]. The loss of orientation is attributed to local stress/strain [3] induced by the lattice mismatch [6] during deposition and/or large substrate surface roughness [6,9]. These characteristics, combined with the thermodynamic instability of (Al,Sc)N, are thought to promote Sc segregation to the grain boundaries, further accelerating phase separation and/or loss of orientation [3,10]. In addition, [001]-oriented hcp α-Ti or [111] rock-salt TiN may constitute a better seeding layer than fcc or bcc metals, since these Ti-based materials can provide smaller epitaxial mismatch to (Al,Sc)N. However, earlier studies of <1 μm thick sputtered undoped AlN films reported that fcc metal seeding layers resulted in well textured films, while bcc or hcp lattices did not [6]. On the other hand, refs. [7,11,12] found that α-Ti and TiN are indeed suitable seeding layers for AlN or (Al,Sc)N films. The large variety of parameters controlling the outcome of reactive sputtering deposition of Sc-doped AlN, e.g., Sc concentration, number of sputtering targets, time, temperature, substrate, seeding layer(s), Ar/N_2_ plasma gas pressure, and volume ratio can largely account for the broad range of (well-oriented) film thicknesses and strength of electromechanical coupling that have been reported [12,13,14,15,16].

The present work proposes replacing a chemically inert seeding layer with TiN/α-Ti. Our procedure takes advantage of the observation that DC sputtering of Ti metal produces well [001]-oriented films on a variety of substrates [17,18,19]. However, while [001]-textured α-Ti presents ~5.1% lattice mismatch to (Al,Sc)N, this is reduced to ~3.7% for the case of [111]-oriented TiN. (See Supplementary, Section S1). In the following, we provide evidence for the in situ formation of TiN films with thickness of several nm during reactive sputtering of Al_1−x_Sc_x_N, (x = 0.25) with nitrogen plasma at the surface of a deposited [001]-textured α-Ti layer. The proposed method leads to stable, piezoelectric [001]-textured films with thickness up to 3 µm.

## 2. Materials and Methods

Six N purity gases (N_2_, argon, O_2_) were used, supplied by Gas Technologies, Israel. Hydrofluoric acid (HF), organic solvents, acetone, and isopropyl alcohol (IPA) were semiconductor grade (CMOS, Sigma Aldrich, St. Louis, MO, USA).

### 2.1. Deposition of Titanium Films

First, 50 nm-thick titanium films were deposited by DC sputtering for 10 min while maintaining the substrate at room temperature. Two-inch diameter substrates were used: (100) p-Si silicon wafers (10–30 ohm·cm, University Wafers, thickness 250 ± 25 µm) and D263 borosilicate glass (SCHOTT, thickness 500 ± 50µm). Substrates were cleaned with solvents in order to increase polarity: acetone, isopropyl alcohol, deionized water. Diluted (4 vol%) HF was then used to remove the native oxide layer as well as surface contaminants. The substrates underwent argon and oxygen plasma cleaning to remove organic contaminants in the sputtering chamber at 10 mTorr pressure with oxygen/argon volume ratio of 1:1. The Ti films were deposited from a 2-inch diameter, 5N purity Ti target, (Abletarget, Beijing, China) by DC magnetron sputtering (ATC Orion Series Sputtering Systems, AJA International, Inc., Scituate, MA, USA) with power level 150 W. The distance between the magnetron and the substrate was 24 cm; the pressure of Ar in the chamber during deposition was 5 mTorr. For the study of the diffusion of nitrogen into the surface of the α-Ti films, they were exposed to nitrogen plasma at 5 mTorr pressure for 30 min at 673 K, using the AJA glow discharge option.

### 2.2. Deposition of Al_0.75_Sc_0.25_N Thin Films

Al_0.75_Sc_0.25_N films were deposited by DC reactive sputtering from Al_0.75_Sc_0.25_ metal alloy targets onto the Ti-seeding layers prepared as described above. Then, 250 W power was applied to a 3-inch diameter magnetron loaded with 5N purity metal alloy targets (Abletarget, Beijing, China). The pressure in the chamber was 5 mTorr and the volume ratio between argon and nitrogen was 1:4. The samples listed in Table 1 were prepared with a deposition temperature profile of 30 min at 673 K followed by sputtering for 8–13 h at 523 K.

### 2.3. Measurement of the Piezoelectric Coefficient and Pyroelectric Response

The resulting films were covered with a 50 nm thick titanium layer, which served as the top electrode. Wafers with [substrate\Ti\Al_0.75_Sc_0.25_N\Ti] film stacks were cut into rectangular (1 cm wide and 2–4 cm long) plates. These were mounted as cantilevers in a deflection monitoring setup [20] and bias was applied between the top and bottom Ti layers. The piezoelectric coefficient was calculated from the stress induced in the cantilever due to voltage application. The stress was calculated using the Stoney formula under the assumption of purely cylindrical bending (zero Gaussian curvature). The pyroelectric response was measured with the (Chynoweth) periodic temperature change method using a modulated IR laser (wavelength 1560 nm, 12 W/cm^2^ OSTECH, Berlin, Germany) operating at 17 kHz [21,22,23]. In order to ensure maximum radiation absorption, the 2 mm diameter Ti contacts prepared for these measurements were covered with carbon black.

### 2.4. Film Characterization—SEM, AFM, EDS, XRD, XPS

Film thickness was measured on sample cross-sections with a scanning electron microscope SEM (Zeiss Sigma 500, and Zeiss Supra 55VP SEMS, 4–8 keV). SEM images were also used to estimate mean grain size and morphology of both the surface and cross-section. Nanoscale topography maps were acquired with an atomic force microscope—Multimode AFM (Bruker, Goleta, CA, USA) in the peak-force tapping mode, using a PNP-TRS probe (NanoAndMore Wetzlar, Germany). Elemental analysis was performed by energy dispersive X-ray spectroscopy (EDS) using a four quadrant detector (Bruker, FlatQUAD) installed on the Zeiss Ultra 55 SEM. Accelerating voltage was 8 kV. X-ray diffraction (XRD) patterns were collected with a TTRAX III diffractometer (Rigaku, Tokyo, Japan) in Bragg-Brentano mode. To characterize film texture, pole figures were recorded at the relevant Bragg angle using a Euler cradle plus a Shultz slit for limiting the footprint of the extended X-ray illumination spot arising from sample tilt. The intensity and line width of the Al_0.75_Sc_0.25_N (002) diffraction peak monitored the quality of c-axis texture. The diffracted intensity of (100) and (011) pole figures was too weak to be detected, leading to an estimate that they are at least 500 times weaker than the (002) peak. Film in-plane stress was deduced from the change in the wafer curvature, before and after film deposition, using a DektakXT stylus profilometer. Since the Al_0.75_Sc_0.25_N film was by far the thickest in the stack, in-plane stress was calculated neglecting the mechanical properties of other layers. X-ray photoelectron spectroscopy (XPS, Kratos AXIS-Ultra DLD spectrometer with monochromatic Al K_α_ source at low power, 15–75 W) was used for surface chemical analysis as a determinant of TiN layer formation. Independently, XPS was used as a non-contact probe of the Al_0.75_Sc_0.25_N layer pyroelectric response [24], for which the sample temperature, (RT or when cooled by liquid nitrogen, (233–283 K)), was monitored by a thermocouple located in close proximity to the back side of the sample. Repeated scans were made at each temperature in order to accurately quantitate binding energies upon temperature stabilization at the surface. The sign of the pyroelectric coefficient and the temperature dependence of a relevant XPS peak (in our case N 1s) are both reliable monitors of the film polarity. [21,24,25,26]. These measurements are not sensitive to details of the material surface, and consequently they are the methods of choice in this report.

## 3. Results

### 3.1. Formation of TiN on a Ti Seeding Layer

**Figure 1:**
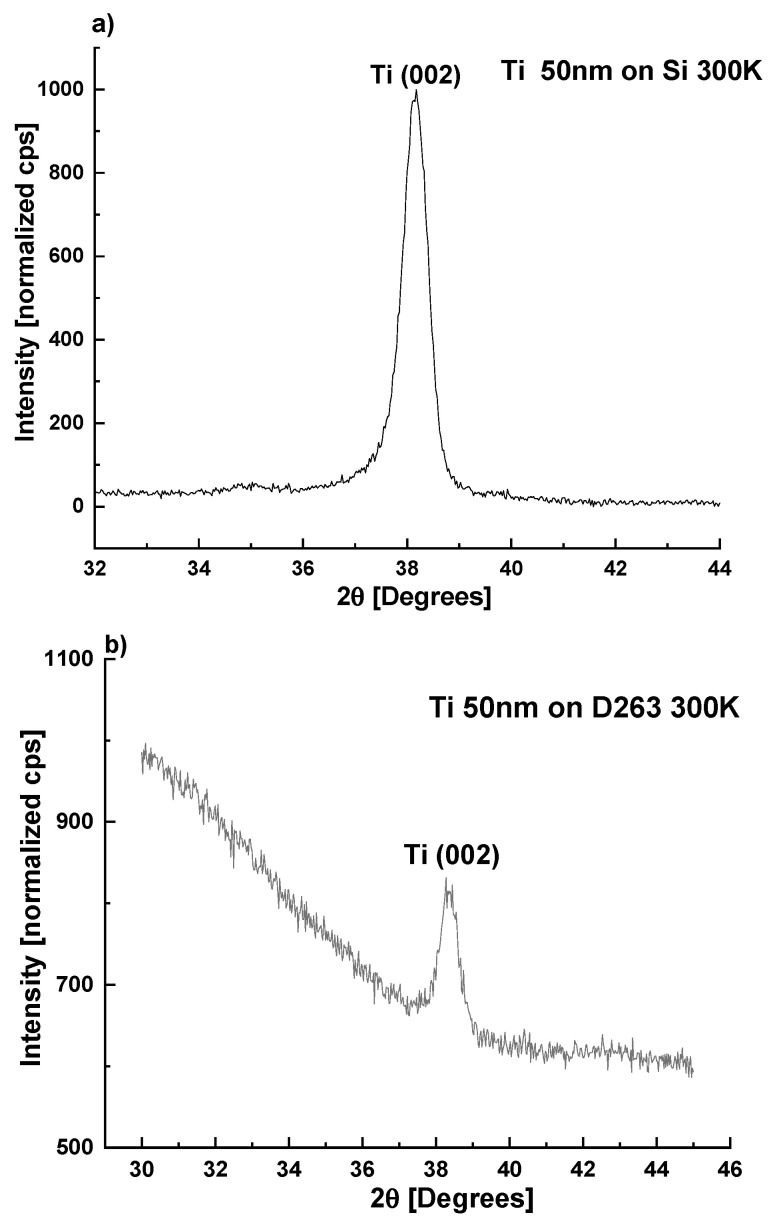
XRD patterns of 50 nm-thick layers of Ti deposited at 300 K on (**a**) (100) Si wafers and (**b**) D263 borosilicate glass, demonstrating strong [001] texture.

Sputtered Ti is known to grow as α-phase (hcp) with preferred [001] orientation [17,18,19]. Further, 50 nm-thick Ti films were deposited as a seeding layer by DC magnetron sputtering on both types of substrates as described above (Section 2.1). According to the XRD patterns (Figure 1), the films are indeed α-Ti with [001] texture: the (002)-diffraction peak dominates the diffraction patterns with peak width Δ2Θ ~0.55° for both substrates.

**Figure 2:**
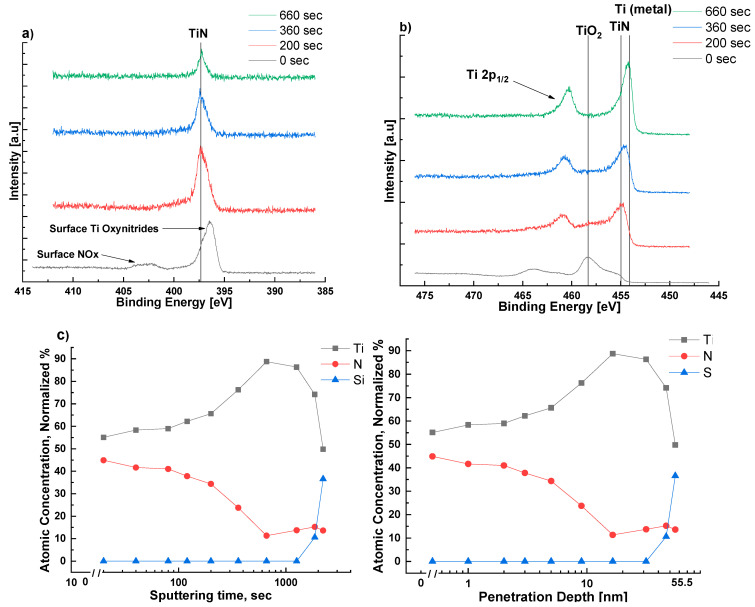
XPS elemental scans of nitrogen N 1s (**a**) and titanium Ti 2p (**b**) from the surface of a 50 nm titanium layer deposited on a (100) silicon substrate, and subsequently exposed to nitrogen plasma at 673 K; (**c**) Normalized atomic concentrations of N, Ti, and Si (total 100%) as a function of argon sputtering time in the XPS chamber; the sputtering rate is estimated to be ~$2.5\times 10^{-2}\frac{\;nm}{\sec}$. Using this sputtering rate, an equivalent graph of atomic concentrations of Ti and N as a function of depth from the surface is also included in (**c**).

To investigate the putative formation of [111] oriented TiN on the α-Ti seeding layer (epitaxial relationships are presented in Supplementary Figure S1), sputtered Ti films were exposed to a glow discharge nitrogen plasma at 673 K for 30 min. Under these conditions of time and temperature, XRD could not detect the presence of TiN. (see Supplementary Section S2 for more successful alternative sputtering time and temperature). However, the film surface was then investigated with XPS: peaks at 396 eV and 400 eV (both N 1s) and 457 eV (Ti 2p), which can be assigned to oxidized TiN [27,28], were observed. Oxidation presumably occurred during transfer of the films from the sputtering system to the XPS chamber. Following removal of ~1 nm from the surface layer via argon sputtering within the XPS chamber, these peaks were ‘replaced’ by those associated with TiN at binding energies 397 eV (N 1s) and 455 eV (Ti 2p) [27,28,29]. With extended argon sputtering time, a gradual transition from TiN to metallic Ti was observed, until the Si substrate was exposed. From the calculated profile of nitrogen atomic concentration vs. time (Figure 2c), we estimate the TiN layer, formed upon exposure of Ti to nitrogen plasma, to be significantly less than 10 nm thick. This thin layer is likely created via nitrogen diffusing into the Ti metal, similar to industrial plasma nitriding [30,31].

**Figure 3:**
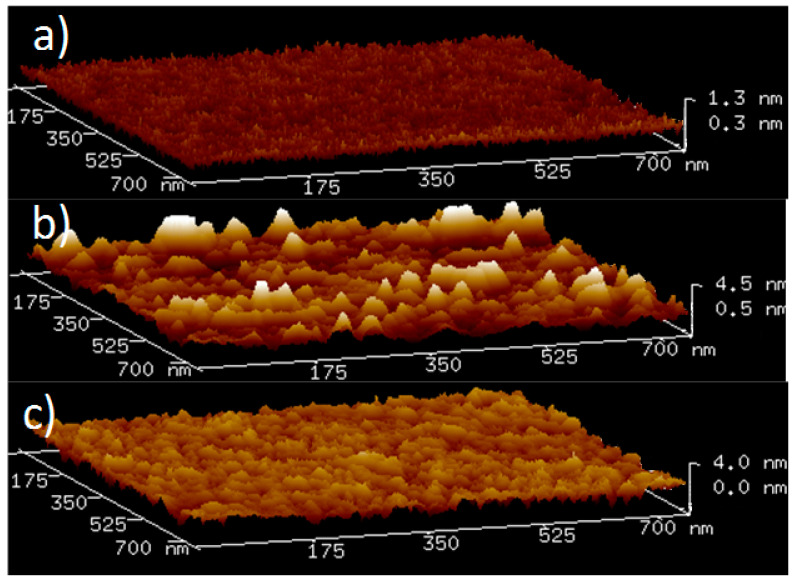
AFM images of (**a**) a (100) silicon wafer following cleaning procedures as described in the Materials and Methods section; (**b**) 50 nm-thick Ti film deposited on the wafer at 300 K; (**c**) the same film following exposure to $N_{2}$ plasma at 673 K for 30 min.

AFM images revealed significant smoothing of the Ti layer surface upon formation of TiN (Figure 3). The root mean square roughness of the as-deposited Ti films over an area of 750 × 750 nm^2^ was 1.1 nm; reaction with nitrogen plasma reduced the roughness to 0.68 nm, which is favorable for the growth of textured (Al,Sc)N [6].

### 3.2. Reactive Sputtering of AlScN

**Figure 4:**
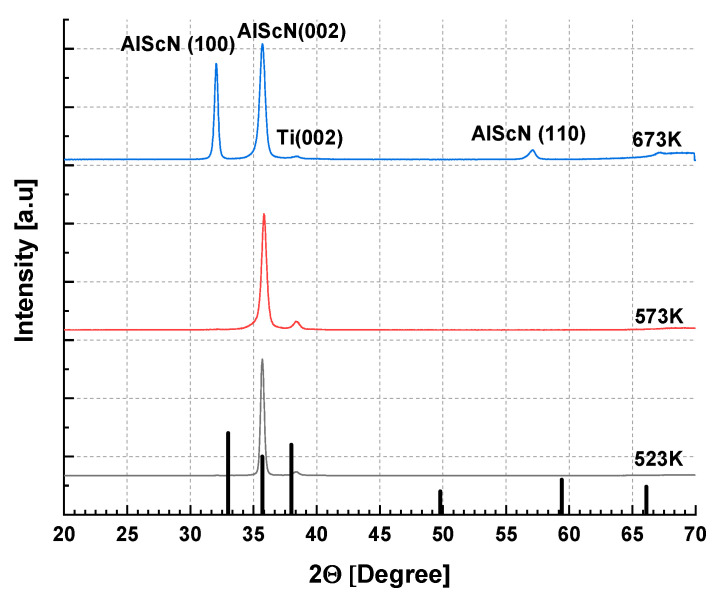
XRD patterns measured in Bragg–Brentano geometry of Al_0.75_Sc_0.25_N films grown on Si (100) wafers, covered with a Ti seeding layer, according to reactive sputtering protocols with nitrogen plasma described in the Materials and Methods section of the main text. The (002) peak becomes progressively narrower as the temperature is lowered: 673 K (blue trace); 573 K (red trace); or 523 K (black trace). Vertical lines mark positions and relative intensities of major AlN powder diffraction peaks: 100, 002, 101 [32].

**Figure 5:**
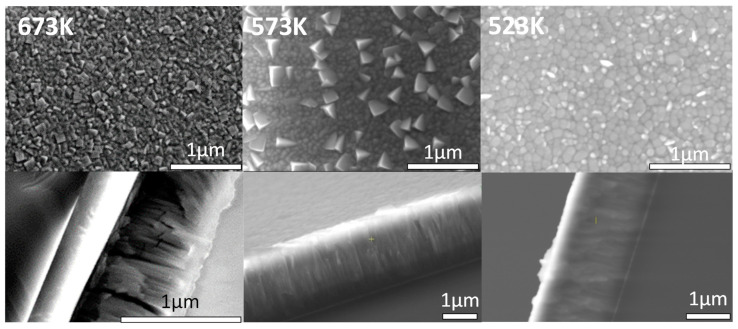
Top view and cross-section SEM images of 2 µm-thick Al_0.75_Sc_0.25_N films deposited on Ti coated Si(100) wafers during ~8 h of reactive sputtering at the indicated substrate temperatures as described above.

Thin films of Al_0.75_Sc_0.25_N were deposited as described in Section 2.2 on both (100) Si wafers and D263 glass substrates coated with 50 ± 10 nm [001]-textured Ti. Without breaking the vacuum after the deposition of Ti seeding layers, the substrates were then heated in the sputtering chamber to 673 ± 10 K. Reactive DC sputtering from a metallic alloy target, Al_0.75_Sc_0.25,_ was performed for 30 min in the nitrogen/argon plasma (see Section 2.2). Sputtering then continued for ~8 h at either 673 K, 573 K, or 523 K. At 673 K, the highest deposition temperature employed, several (Al,Sc)N diffraction peaks were observed by XRD, while in SEM images, pyramidal grains were present on the sample surface. Lowering the final deposition temperature to 523 K resulted in preferred [001] (Al,Sc)N texture as observed by XRD [Figure 4] as well as reduction in the number of abnormally oriented grains in SEM images [Figure 5].

**Figure 6:**
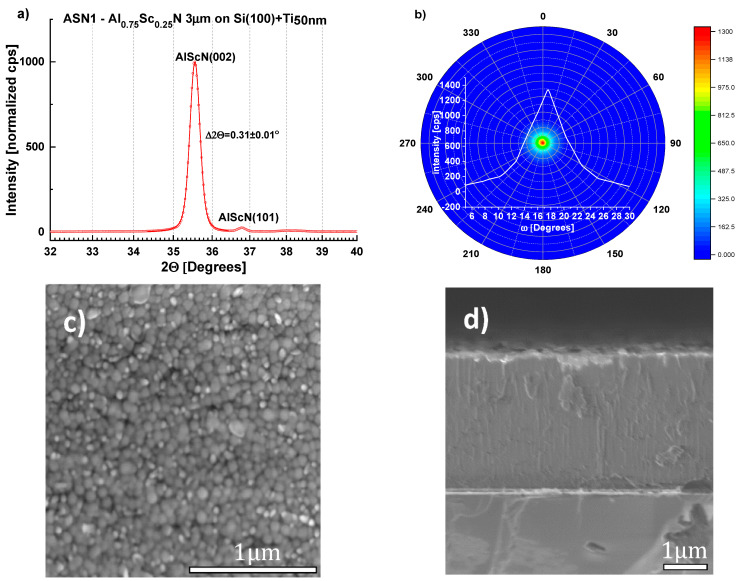
Structural characterization of sample ASN1 (see Table 1) (**a**) XRD pattern showing the (002) Bragg-peak at 2θ = 35.55°, Δ2θ  = 0.31 ± 0.01°; (**b**) (002) pole figure and equivalent rocking-curve (generated from superimposed pole figure cross-sections each with a different value of the in-plane rotational angle, beta.) Gaussian fits to the ω-scans for ASN1 on three different Si wafers give FWHM ≈ 5.0 ° with standard deviation of ±1°; (**c**,**d**) SEM images of the surface and cross-section of sample ASN1 showing pebble-like grains at the surface (mean transverse dimension, 84 nm, as determined by the lineal intercept method) and columnar growth, respectively.

**Figure 7:**
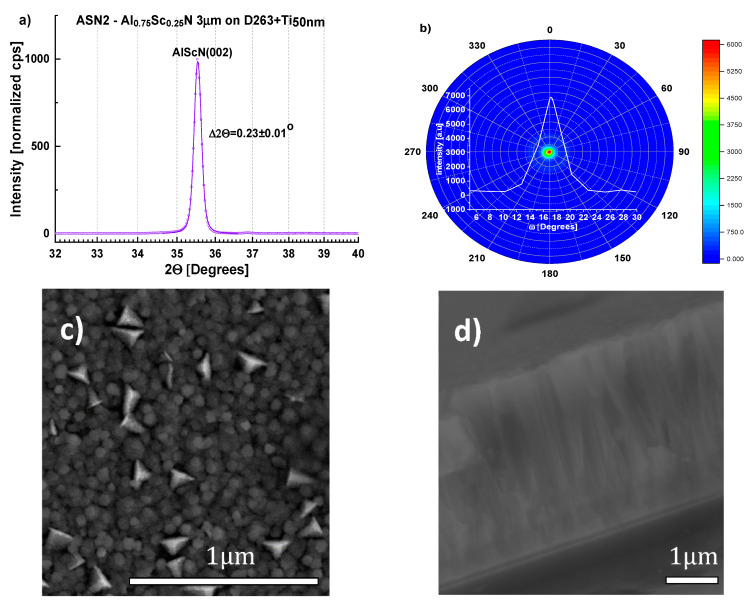
Structural characterization of sample ASN2 (see Table 1). (**a**) XRD pattern showing the (002) Bragg peak at 2θ = 35.54°, Δ2θ = 0.23 ± 0.01°; (**b**) (002) pole figure and equivalent rocking-curve (generated from superimposed pole figure cross-sections as described for Figure 6b); (**c**,**d**) SEM images of the surface and cross-section showing pebble-like grains at the surface, mean transverse size 101 nm (as determined by the lineal intercept method) and uniform columnar growth, respectively. Misoriented grains are more prevalent in the SEM image of ASN2 as compared to ASN1.

**Figure 8:**
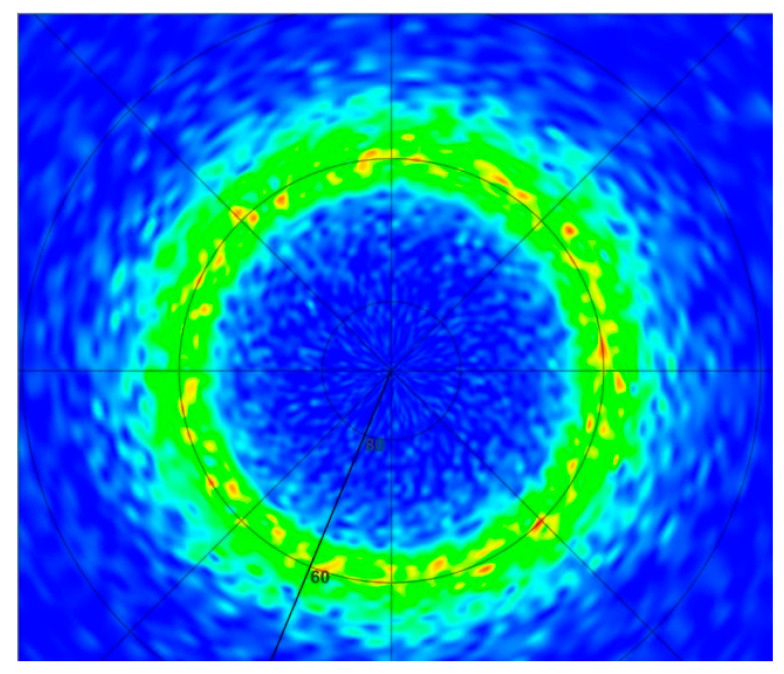
Ring-like intensity distribution for the (103) pole figure of ASN1.

Consequently, following an initial 30 min of sputtering at 673 K, samples (ASN1-3) were deposited at 523 K for 8–13 h at 3.5 to 4 nm/min depending on the desired film thickness (2–3 µm). Irrespective of the substrate, (100)-Si or D263 borosilicate glass, films up to 3 µm thick were produced (Table 1, Figure 6 and Figure 7). Although metal stoichiometry of the deposited films may differ from that of the alloy target, EDS showed negligible change, i.e., Al_0.75_Sc_0.25_ (see also Supplementary Section S3). The XRD patterns of the Al_0.75_Sc_0.25_N films were dominated by a strong (Al,Sc)N wurtzite (002) diffraction peak at 2θ≈ 35.5°, peak width Δ2θ ≈ 0.31 ± 0.01°. Pole figures collected for this peak from films grown on both substrates (Figure 6a,b and Figure 7a,b) attest to c-axis texture. We note that the ring-like intensity distribution of the pole figure measured for the (103) diffraction peak further supports this claim (Figure 8). By fitting a Gaussian profile to a superposition of (002) pole figure cross-sections with different values of the in-plane rotational angle, beta, an equivalent rocking curve (ω-scan) is generated. Gaussian FWHM ≈ 5.0° with standard deviation ±1° for 3 μm (Al,Sc)N films on three different Si wafers and on D263 is obtained (Figure 6b and Figure 7b). (100), (101) diffraction peaks were of negligible intensity [32]. Diffraction characteristic of phase-separated ScN (rock-salt) was not detected [33].

**Figure 9:**
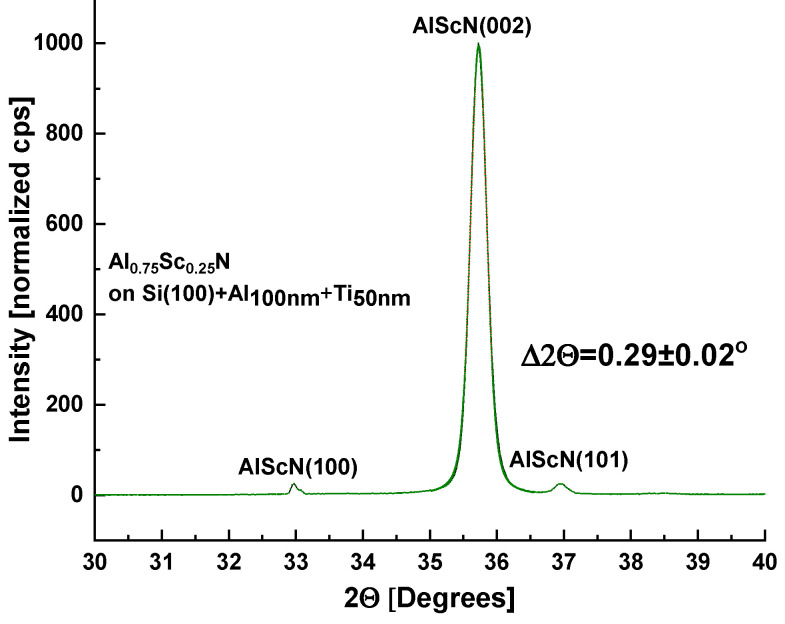
XRD pattern of sample ASN3 grown on (100) Si with a Ti seeding layer and with a stress-relieving Al layer (see Table 1).

**Figure 10:**
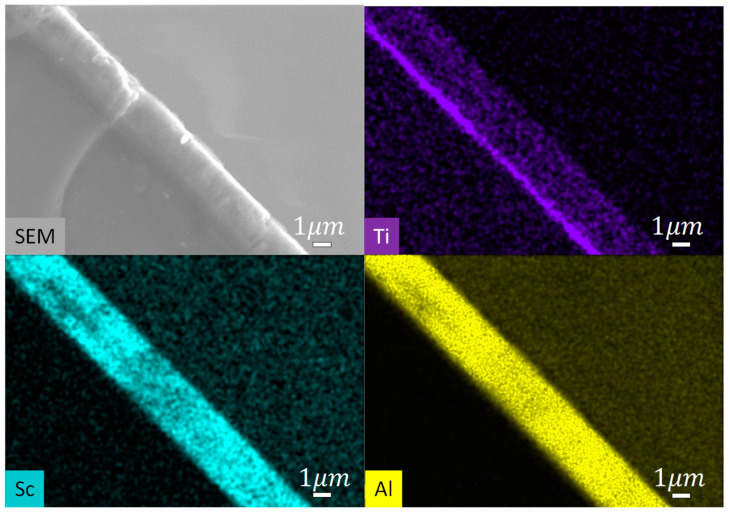
SEM image and elemental mapping of a cross-section of a Al_0.75_Sc_0.25_N thin film deposited on a Si wafer covered with a Ti/TiN seeding layer. All scale bars designate 1 µm. Electron beam energy during data acquisition was 8 keV.

Scanning electron microscope (SEM) images of the film surfaces show pebble-like grains with mean transverse dimension ~85–100 nm (Figure 6c, Figure 7c). For five Si wafers, surface contamination and misoriented, pyramidal grains occupy < 6% of the area as determined from replicate measurements. It was found that film quality was not modified by the presence of a 100 nm-thick Al layer introduced to promote relaxation of the compressive stress typical of sputtered (Al,Sc)N [34,35,36] (Figure 9). Compressive stress in all films, with or without an Al stress relaxation layer, was <100 MPa, as calculated from the change in wafer curvature, using the Stoney formula (Table 1), indicating that the Ti\TiN seeding layer supports low deposition stress. EDS elemental mapping demonstrates homogenous distribution of Al and Sc, with no indication of ScN segregation (Figure 10).

### 3.3. Pyroelectric Measurements

**Figure 11:**
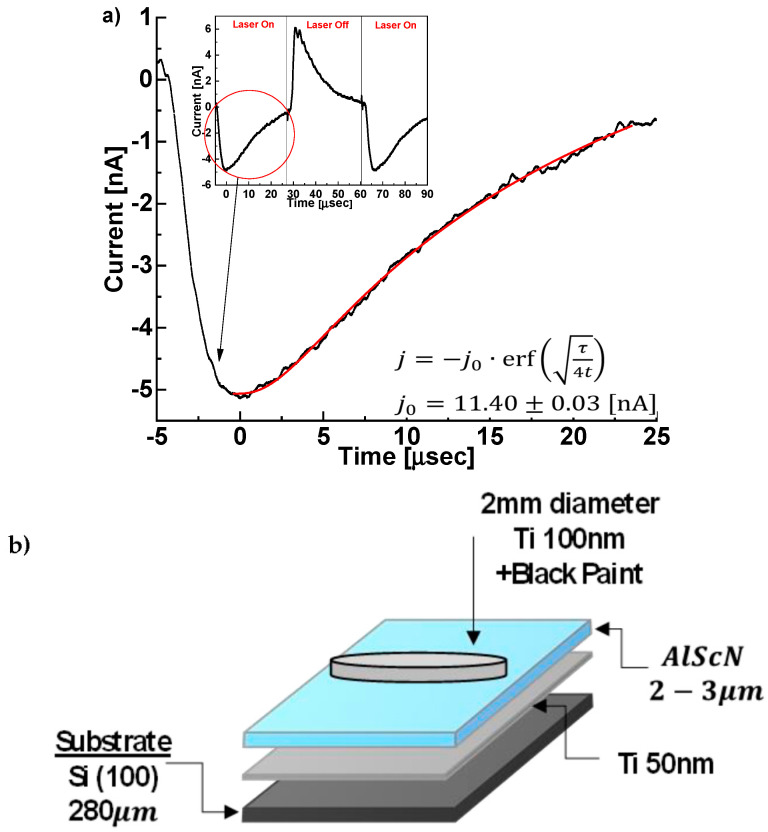
(**a**) Pyroelectric current *j* in sample ASN1 periodically heated with an IR laser as described in the Materials and Methods section. Inset shows the heating phase of the current decay which was used for fitting to the error function. The pyroelectric response is calculated from $j_{0}$. [22,23]. (**b**) Schematic of sample ASN1 as prepared for these measurements with a 2 mm diameter, black paint-coated upper Ti electrode.

**Figure 12:**
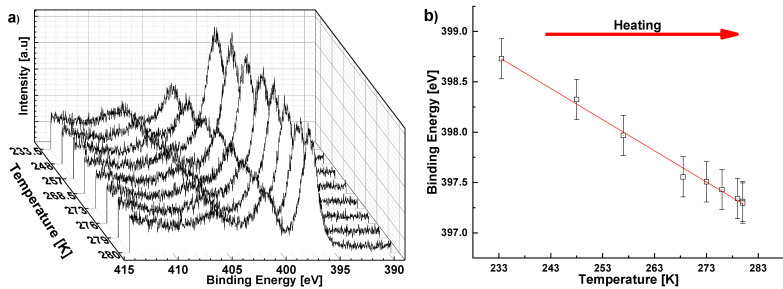
(**a**) XPS measurement of the N 1s spectrum at different temperatures (233–280 K) of the ASN1 sample; (**b**) N 1s peak positions as a function of temperature during heating in ultra-high vacuum. The monotonic shift to lower binding energies upon heating indicates electron accumulation, which is consistent with Al-termination [24]. Error bars indicate experimental uncertainty.

To determine whether the polar axis is directed toward, or away from, the substrate in sample ASN1, i.e., to distinguish between [001] and [00$\bar{1}$] orientations, we measured the pyroelectric effect with PTC [21,25] and with XPS [24,37,38]-based methods. The PTC measurements revealed that the film pyroelectric coefficient is $\alpha_{f}=-13.9\pm 0.1\;\left[\mu C/m^{2}K\right]$ (Figure 11; and Supplementary Section S4) [25], which is close to that previously reported for this composition (25% Sc) [39]. However, in our case, $\alpha_{f}$ is negative. In the XPS measurements, the N 1s peak shifts to lower energies reversibly upon heating (Figure 12), providing additional support for the negative pyroelectric response. The sign of the pyroelectric response suggests that the films are [001] oriented, i.e., the top surface is Al-terminated. This is in contrast to previous reports of films grown on inert metallic seeding layers, all of which are N-terminated [40,41,42]. On the other hand, the existence of Al-terminated films is consistent with the proposed nucleation mechanism, i.e., the epitaxial growth of (Al,Sc)N wurtzite structure begins from an N-terminated face of TiN.

### 3.4. Measurement of the Piezoelectric Coefficient

**Figure 13:**
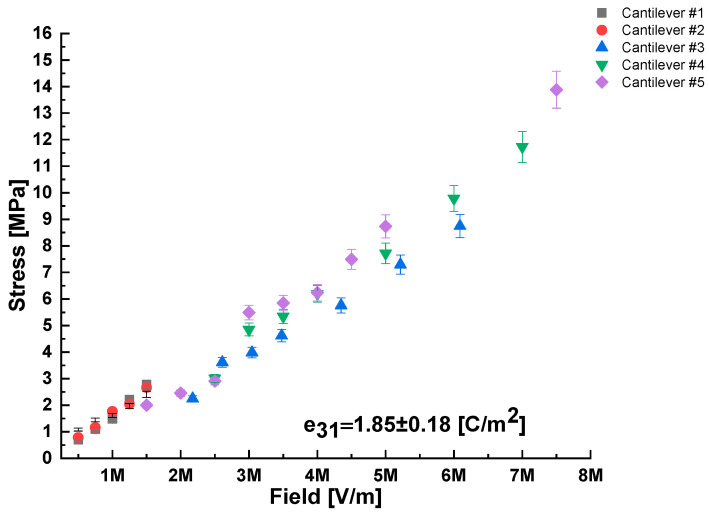
Dependence of quasi-static (0.1 Hz) stress vs. electric field for five cantilever samples, fabricated on three different Si wafers, as quantified by cantilever deflection in response to the electric field applied perpendicular to the plane of the cantilever, along with knowledge of thickness and mechanical properties of the AlScN covered Si wafers.

The current detected during the piezoelectric response measurements was very small (1–20 nA), evidence that the cantilevers were not shorted. The mean converse transversal piezoelectric coefficient calculated from the cantilever deflection amplitude and averaged over five cantilevers was $e_{31}=1.85\pm 0.18\;C/m^{2}$ (Figure 13 and Table 2). This value is similar to those reported in the literature for films with the same Sc concentration (25 mol% Sc) but <1 µm thick [43]. Thus, our preparation protocol and larger film thickness do not cause deterioration of the piezoelectric coefficient. Similarly, the dielectric permittivity of the AlScN films, as measured by impedance spectroscopy at 0.5 Hz, 0.1 V_ac_, is ε_r_ =12.5±1.0 in agreement with refs. [43,44]. We note that the larger than usual film thickness (>2.5 µm), and the ability to withstand at least 7.5 MV/m electric field, implies that the films can deliver >$1.85\pm 0.18\;C/m^{2}$ × 7.5 MV/m × 2.5 µm = 33 J/$m^{2}$ of elastic energy per unit area [45]. The practical importance of film thickness for some MEMS applications is made clear by considering Stoney’s formula in the form: $\delta_{max}=\sigma_{max}h_{f}3L^{2}\left(1-\nu_{s}\right)/E_{s}h_{s}\;$ [46], where *h_f_* is the film thickness; $\sigma_{max}$ is the maximum stress sustainable in the film before failure; $\delta_{max}$ the maximum deflection of the cantilever; *L* the cantilever length; *E_s_* the Young’s modulus; *h*_s_ is the thickness; and $\nu_{s}$ the Poisson ratio, all relating to the substrate on which the cantilever is fabricated. One can see that for a fixed cantilever length, the maximum deflection is proportional to the film thickness (*h_f_*). Applications requiring large values of elastic energy density include microfluidics and tunable capacitors.

## 4. Summary

In summary, a protocol for successfully depositing stable, [001] textured, 2–3 µm thick films of Al_0.75_,Sc_0.25_N, is described. The procedure exploits the formation of sputtered, α-phase (hcp) Ti thin films with [001]-texture. We present evidence that chemical reaction between [001]-textured α-Ti and nitrogen plasma during reactive sputtering of (Al,Sc)N leads to the rapid formation of an interfacial, very thin TiN seeding layer, presenting lattice mismatch of only 3.7% between the (111) planes of TiN and the (001) planes of the Al_0.75_,Sc_0.25_N film. This is up to three times smaller mismatch than that presented by inert metal seeding layers commonly used for film preparation. We suggest that it is this close lattice match that is an important determinant for the low values of deposition compressive stress, *σ_f_* < 100 MPa, maintained in spite of the thickness of the (Al,Sc)N film. In contrast to earlier reports, the Al_0.75_,Sc_0.25_N films prepared in the current study are polar with [001] orientation rather than [00$\bar{1}$]: this is consistent with growth initiation on a nitrogen layer. Although 2–3 µm thick, these films display pyroelectric and piezoelectric coefficients similar to those reported for significantly thinner films (<1 µm) [8,39]. An important practical advantage of the proposed growth technique is its compatibility with a variety of substrates commonly used for actuators or MEMS, as demonstrated here for both Si wafers and D263 borosilicate glass. Since the maximum temperature (673 K) employed is considerably lower than that observed to be detrimental for CMOS chips (798 K), the proposed procedure should be fully compatible with CMOS electronics [47]. Additionally, thicker films can potentially achieve increased piezoelectric stress/strain by permitting application of higher voltage, but without increase in the magnitude of the electric field.

## 5. Patents

A patent application based on the data reported in this article has been submitted and approval is pending.

## Figures and Tables

**Table 1:** Description of the (Al,Sc)N film samples deposited as described in Section 2.2. The Stoney formula was used for calculation of residual in-plane film stress. $\sigma =\frac{E_{s}h_{s}^{2}}{6\left(1-\nu_{s}\right)h_{f}}\left(\frac{1}{R}-\frac{1}{R_{0}}\right)$, where *E* is the Young’s modulus; *h*, the thickness; *v*, the Poisson ratio; *R*, the cantilever radius of curvature following deposition; and *R*_0_ is the initial radius of curvature. Subscripts *s*, and *f* refer to the substrate and thin film, respectively.

Sample	Substrate	Under-Layer	AlScN *h_f_* [μm]	Film Stress *σ* [MPa]
ASN1	Silicon (100)	50 nm Ti	3 ± 0.1	60 ± 10
ASN2	Borosilicate glass (D263)	50 nm Ti	3 ± 0.1	80 ± 12
ASN3	Silicon (100)	100 nm Al + 50 nm Ti	2 ± 0.1	56 ± 9

**Table 2:** Transversal, converse piezoelectric coefficient e_31_ for AlScN thin films, evaluated for five rectangular cantilevers fabricated on three different Si wafers.

Cantilever #	$e_{31}\left[C/m^{2}\right]$
1	2.01 ± 0.15
2	1.85 ± 0.11
3	1.56 ± 0.10
4	1.82 ± 0.12
5	2.00 ± 0.20

## Data Availability

The data presented in this study are available on request from the corresponding author.

## Supplementary Materials

The following supporting information can be downloaded at: https://www.mdpi.com/article/10.3390/s22187041/s1, Section S1: Epitaxial relationships between hcp [001] oriented Ti; cubic [111] oriented TiN; and hcp [001] oriented AlN; Section S2: XRD profiles and pole figures of textured [001] Ti and [111] TiN on Si; Section S3: EDS elemental analysis of (Al,Sc)N film stoichiometry, sputtered from a single Al_0.75_Sc_0.25_ alloy target; Section S4: Calculating the film pyroelectric coefficient *α_f_* from error function fitting.
