# Supplementary material for: C-Axis Textured, 2–3 μm Thick Al0.75Sc0.25N Films Grown on Chemically Formed TiN/Ti Seeding Layers for MEMS Applications
Source: Sensors (Basel). 2022 Sep 17;22(18):7041. doi: 10.3390/s22187041 (PMC9504120; doi:10.3390/s22187041)
Supplement: Supplementary file 1 [file sensors-22-07041-s001.zip › sensors-1522085-supplementary.pdf]

Supplementary Material

c-axis textured, 2-3μm thick $Al_{0.75}Sc_{0.25}N$ films grown on chemically formed TiN/Ti seeding layers for MEMS applications

Supporting Information

Section S1. Epitaxial relationships between hcp (001) oriented Ti; cubic (111) oriented TiN; and hcp (001*)* oriented AlN .

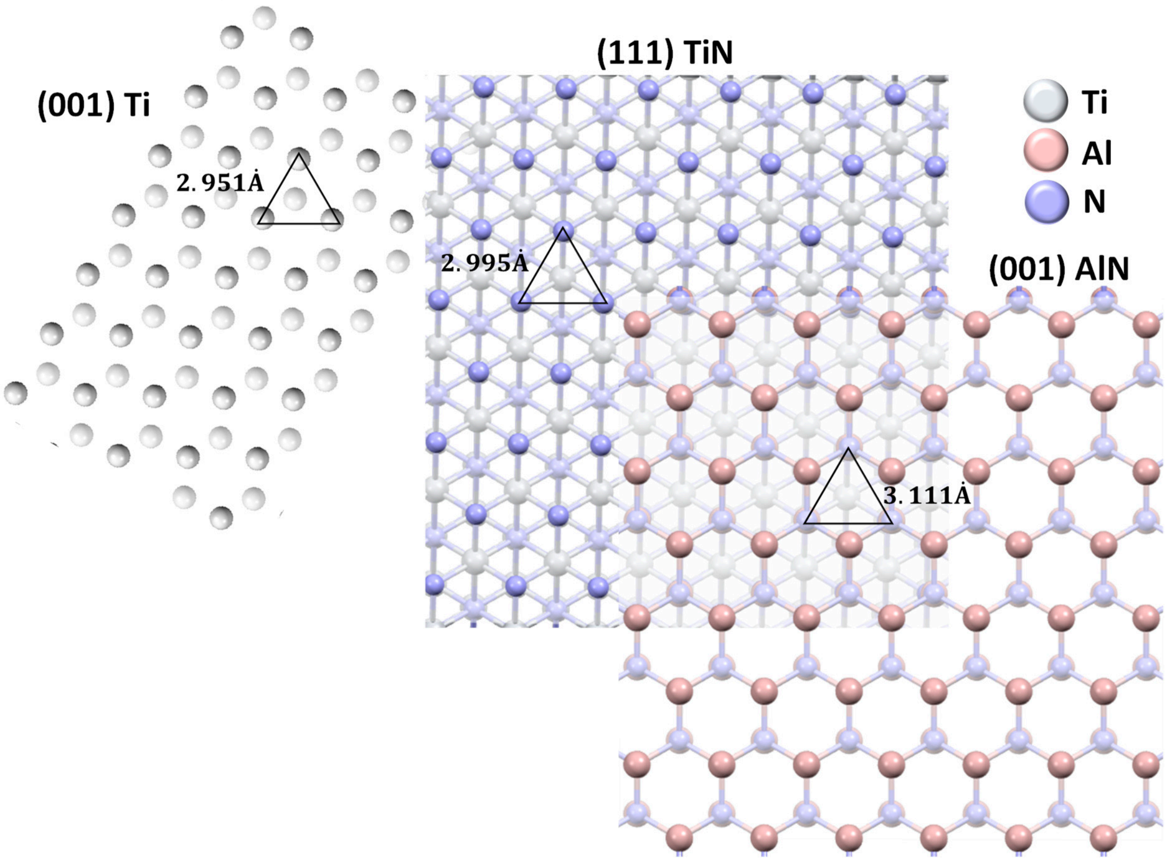

Figure S1. Surface layers presenting small epitaxial mismatch: a Ti(001) plane, comprising equilateral triangles 2.951 Å on a side; a TiN (111) plane, comprising equilateral triangles 2.995Å on a side; Each

TiN nucleation site provides *in-situ* epitaxy for a superimposed AlN (001) plane, comprising equilateral triangles 3.111 Å on a side.

Section S2. XRD patterns and pole figures of textured (001) Ti and cubic (111) TiN.

Following extensive substrate cleaning procedures, as described in Section 2.1 of the main text, 50 nm thick titanium films were deposited by DC sputtering for 10 min, power level 150W, while maintaining the substrate at room temperature. The pressure of Ar in the chamber during deposition was 5 mTorr. Without breaking the vacuum, deposition of Ti continued in the presence of nitrogen/argon plasma (4/1) ratio by volume) for 10 min at 673K, using glow discharge. The substrate temperature was then lowered to 523K and deposition continued for another 10min. TiN films prepared in this way demonstrated preferred (111) orientation ($2\theta = 36.8^{o}$) grown on the underlying Ti(002) film (Figures S2a,b). Pole figure of the corresponding Ti (002) peak, measured on Si wafer substrates, attests to the development of preferred orientation. (Figure S2c) Similar results were reported in [19]

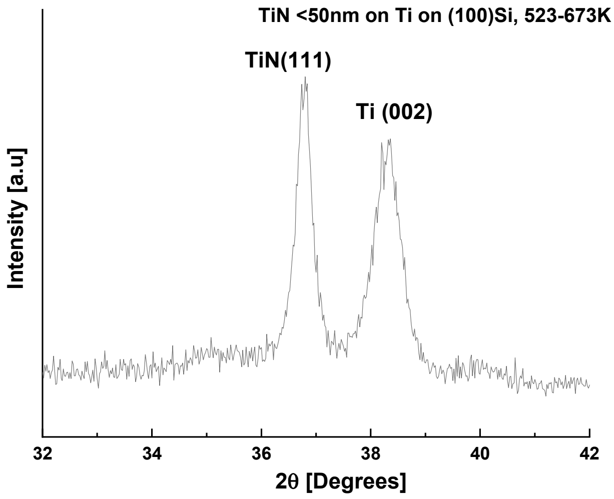

a)

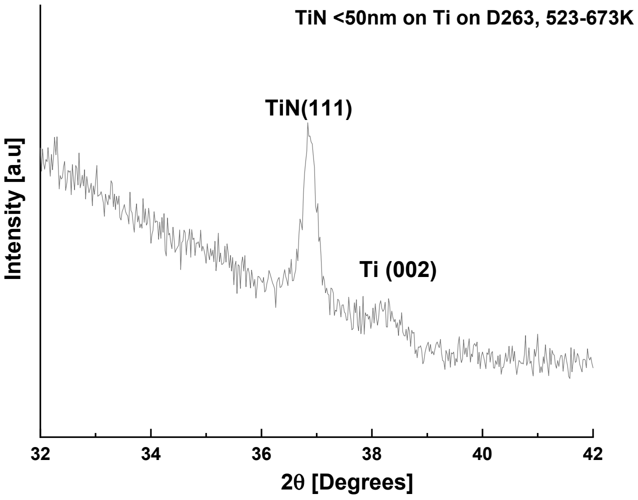

b)

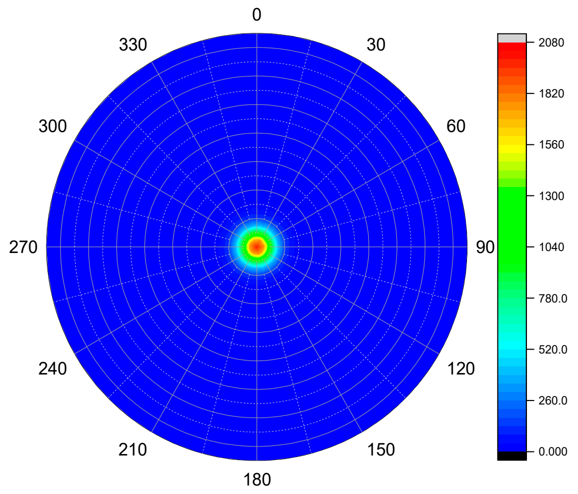

c)

Figure S2. Thin (<50nm) TiN films grown on Ti seeding layers during approx. 20min reactive Ti sputtering in N_2 / Ar plasma , temperature range, 673-523K, demonstrate preferred TiN (111) orientation on Ti (002) grown on Si (a) and D263 borosilicate glass (b) substrates . XRD patterns were measured in Bragg-Brentano geometry as described in the Experimental section. c) Pole figure of Ti(002) on Si , Bragg peak: 2θ=38.35°. Gaussian fit to Ti (002) pole figure cross section, with fixed β, gives FWHM ~ 9° with fitting error ± 1°.

Section S3. EDS elemental analysis of (Al,Sc)N film stoichiometry, sputtered from a single $Al_{0.75}Sc_{0.25}$ alloy target.

Element	Atomic No.	Mass Norm. [%]	Atom [%]	Abs.error [%]
Aluminum	13	63.53	74.4	1.9
Scandium	21	36.47	25.6	1.1

Section S4. Calculating the thin film pyroelectric coefficient α_f from error function fitting

The pyroelectric current $j(t)$ fit to the error function is given by $j(t) = j_0 \cdot \mathrm{erf}\left(\sqrt{\frac{\tau}{4t}}\right)$. The parameter $j_0 = \frac{F_d \alpha_f}{C_v(d_s+\delta)}$, where F_d =12W/cm^2 is the effective laser power applied to the sample; α_f is the film pyroelectric coefficient; c_v is the heat capacity of the Si wafer , 1.64 x 10^6 joules /K• m^2 ; the wafer substrate thickness, $d_s = 280\mu m$; $\delta = 2\mu m$ the thickness of the (Al,Sc)N thin film.
